# Upregulation of Nrf2 and Decreased Redox Signaling Contribute to Renoprotective Effects of Chemerin Receptor Blockade in Diabetic Mice

**DOI:** 10.3390/ijms19082454

**Published:** 2018-08-19

**Authors:** Karla Bianca Neves, Augusto Cesar Montezano, Rheure Alves-Lopes, Thiago Bruder-Nascimento, Rafael Menezes Costa, Roberto S Costa, Rhian M Touyz, Rita C Tostes

**Affiliations:** 1 Department of Pharmacology, Ribeirao Preto Medical School, University of Sao Paulo, Ribeirao Preto 14049-900, Brazil; Rheure.Lopes@glasgow.ac.uk (R.A.-L.); bruderthiago@yahoo.com.br (T.B.-N.); rafael.menezess@yahoo.com.br (R.M.C.); rtostes@usp.com (R.C.T.); 2 Department of Physics and Chemistry, Faculty of Pharmaceutical Sciences of Ribeirao Preto, University of Sao Paulo, Ribeirao Preto 14040-093, Brazil; 3 Institute of Cardiovascular and Medical Sciences, University of Glasgow, Glasgow G12 8TA, UK; Augusto.Montezano@glasgow.ac.uk (A.C.M.); Rhian.Touyz@glasgow.ac.uk (R.M.T.); 4 Department of Pathology and Legal Medicine, Ribeirao Preto Medical School, University of Sao Paulo, Ribeirao Preto 14040-900, Brazil; rscosta@fmrp.usp.br

**Keywords:** chemerin, ChemR23, kidney, type 2 diabetes, oxidative stress

## Abstract

Chemerin, acting through its receptor ChemR23, is an adipokine associated with inflammatory response, glucose and lipid metabolism and vascular function. Although this adipokine has been associated with the development and progression of kidney disease, it is not clear whether the chemerin/ChemR23 system plays a role in renal function in the context of diabetes. Therefore, we sought to determine whether ChemR23 receptor blockade prevents the development and/or progression of diabetic nephropathy and questioned the role of oxidative stress and Nrf2 in this process. Renal redox state and function were assessed in non-diabetic lean db/m and diabetic obese db/db mice treated with vehicle or CCX832 (ChemR23 antagonist). Renal reactive oxygen species (ROS) production, which was increased in diabetic mice, was attenuated by CCX832. This was associated with an increase in Nox 4 expression. Augmented protein oxidation in db/db mice was not observed when mice were treated with CCX832. *CCX832* also abrogated impaired Nrf2 nuclear activity and associated downregulation in antioxidants expression in kidneys from db/db mice. Our in vivo findings highlight the role of the redox signaling and Nrf2 system as renoprotective players during chemerin receptor blockade in diabetic mice. The chemerin/ChemR23 system may be an important target to limit renal dysfunction associated with obesity-related diabetes.

## 1. Introduction

Chemerin, also known as tazarotene-induced gene-2 protein or retinoic acid receptor responder protein-2 (RARRES2), is a 16 kDa protein predominantly secreted by adipose tissue and skin [1,2]. It has been associated with inflammatory response, glucose and lipid metabolism [3] and vascular function [4,5]. Chemerin is a ligand for the chemokine-like receptor 1 (CMKLR1; also called ChemR23), a G_i_ protein-linked receptor mainly expressed by dendritic cells, macrophages, adipose tissue, vascular smooth muscle and endothelial cells, in addition to the chemokine receptor like 2 (CCRL2) and G-protein-coupled receptor 1 (GPR1) [6,7,8].

Chemerin gene expression is elevated in psoriasis [9], inflammatory diseases including ulcerative colitis and Crohn’s disease [10], obesity [11,12,13], and a meta-analysis data linked elevated plasma levels of chemerin to the metabolic syndrome (MetS) [14]. Pregnant women with preeclampsia also present increased serum levels of chemerin, which is maintained six months after the pregnancy [15]. Enhanced expression of chemerin in white adipose tissue, skeletal muscle, and liver has been reported in db/db mice, an experimental model of obesity-related type 2 diabetes (T2D) where this adipokine exacerbates glucose intolerance, lowers serum insulin levels, and decreases tissue glucose uptake [16,17].

High circulating levels of chemerin are also associated with progression of kidney dysfunction [18] and chronic inflammation, insulin resistance, and disorders of glucose and lipid metabolism, which are abnormalities commonly observed in chronic kidney disease (CKD) [19,20]. In patients with severe renal damage, this adipokine is expressed in tubular epithelial cells and lymphatic endothelial vessels and has been identified as a product of endothelial and proximal tubular epithelial cells. Additionally, the chemerin/ChemR23 system recruits dendritic cells within the kidney and may play a role in renal inflammation [21]. Moreover, it has been demonstrated that chemerin is associated with impaired cardiovascular function [4,5], which may be an aggravating factor to the enhanced cardiovascular risk in CKD.

Several studies have shown that oxidative stress is of major importance in the pathogenesis and progression of renal and systemic diseases [22,23,24,25]. An imbalance in reactive oxygen species (ROS) levels is an outcome of their dysregulated production and/or a deficient antioxidant function [26]. ROS are mainly produced by nicotinamide adenine dinucleotide phosphate (NADPH) oxidases (Nox), which are an important source of superoxide anion (O_2_^−^) and hydrogen peroxide (H_2_O_2_) in the cardiovascular system [27]. Even though multiple Nox homologues have already been identified, Nox 4 is particularly important in renal pathobiology since is the most abundant Nox isoform in the kidney [28], being considered the major source of ROS in renal cells and tissue in diabetes [29]. Diabetic mice commonly exhibit increased vascular and renal oxidative stress, and treatment with antioxidant ameliorates renal disorders in these animals [30,31]. The nuclear erythroid-2 like factor-2 (Nrf2) transcription factor is the master regulator of the endogenous antioxidant defense system [32]. Chemerin, through Nox-derived ROS, stimulates mitogenic and pro-inflammatory signalling pathways promoting vascular damage and remodelling [33].

Although chemerin has been associated with insulin resistance in cardiomyocytes and skeletal muscle cells [34,35] and with kidney disease [18,19], the role of the chemerin/ChemR23 system in renal function in the context of diabetes has not been fully elucidated. We previously showed that increased plasma chemerin is associated with hyperglycemia in obese diabetic db/db mice [36]. Here we extend those studies by exploring the effects of ChemR23 receptor blockade on renal redox homeostasis during the early stages of nephropathy in diabetic db/db mice.

## 2. Results

### 2.1. Chemerin Receptor Blockade Decreases Kidney Mass and Creatinine Levels in db/db Mice

**Figure 1:**
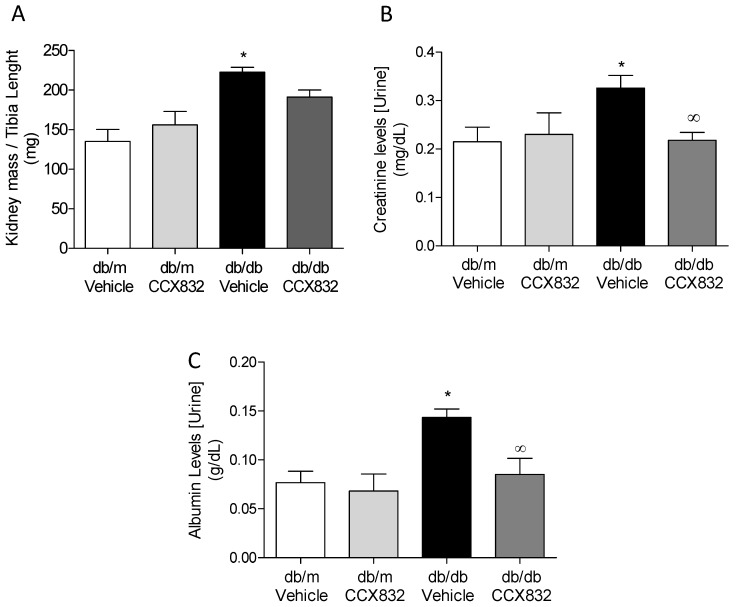
ChemR23 antagonism decreases albuminuria and urinary creatinine levels in db/db mice. Kidney mass (**A**) urinary creatinine, (**B**) and albumin levels, (**C**) in vehicle-treated or CCX832-treated db/m and db/db mice. Kidney mass (g) was normalized by the tibia length (mm). Values represent the mean ± SEM (standard error deviation) of 5 to 8 experiments. * *p* < 0.05 vs. db/m, ∞ *p* < 0.05 vs. db/db vehicle.

In db/db mice, which we previously phenotyped [37,38,39,40] we show that chemerin receptor antagonism by CCX832 did not significantly alter increased kidney mass (Figure 1A), but it decreased urinary creatinine levels (Figure 1B) and albuminuria (Figure 1C) in db/db diabetic mice, indicating that CCX832 prevents renal dysfunction observed in db/db mice. No differences in these parameters were observed between control db/m mice treated with vehicle or CCX832 (Figure 1A–C).

### 2.2. CCX832 Reduces Renal Oxidative Stress in db/db Mice

**Figure 2:**
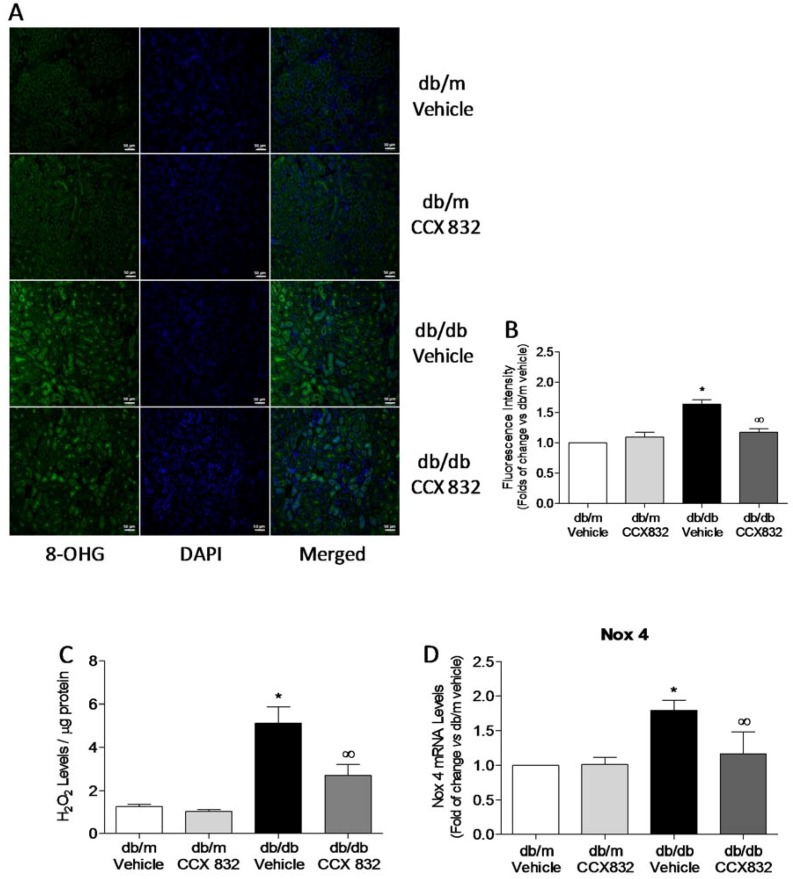
CCX832 reduces renal oxidative stress and Nox 4 expression in db/db mice. (**A**) Representative images and (**B**) quantitative analysis of 8-hydroxyguanosine (8-OHG)-positive nuclei in kidneys from vehicle or CCX832-treated db/m and db/db mice. Scale bar = 50 µm; 20X. (**C**) Renal H_2_O_2_ levels were measured by Amplex red assay. Amplex red values were normalized by protein content. (**D**) Gene expression of Nox 4 in kidneys was determined by real time PCR. The values were normalized by glyceraldehyde-3-phosphate dehydrogenase (GAPDH) gene expression. Results represent the mean ± SEM of 5 to 6 experiments. * *p* < 0.05 vs. db/m, ∞ *p* < 0.05 vs. db/db vehicle.

Considering that chemerin increases ROS production [33], we investigated whether ChemR23 antagonism attenuates renal ROS generation in db/db mice. Figure 2A,B demonstrate that kidneys from obese/diabetic (db/db) mice present increased ROS production in comparison to kidneys from vehicle- and CCX832-treated db/m mice (Figure 2A,B). Renal ROS generation in db/db mice was significantly attenuated by treatment with CCX832 (Figure 2A,B).

Kidneys from db/db mice also exhibited increased H_2_O_2_ levels (Figure 2C), which were associated with increased Nox 4 mRNA expression (Figure 2D). Both outcomes were significantly decreased by ChemR23 antagonism (Figure 2C,D). No differences in H_2_O_2_ or Nox 4 mRNA levels were observed between db/m mice treated with vehicle or CCX832 (Figure 2A–D). No changes were observed in Nox 1 or Nox 2 expression (Supplemental Figure S1). Together, these data suggest that CCX832 ameliorates redox-sensitive processes in kidneys from db/db mice.

### 2.3. CCX832 Reduces Protein Oxidation in Kidneys from db/db Mice

**Figure 3:**
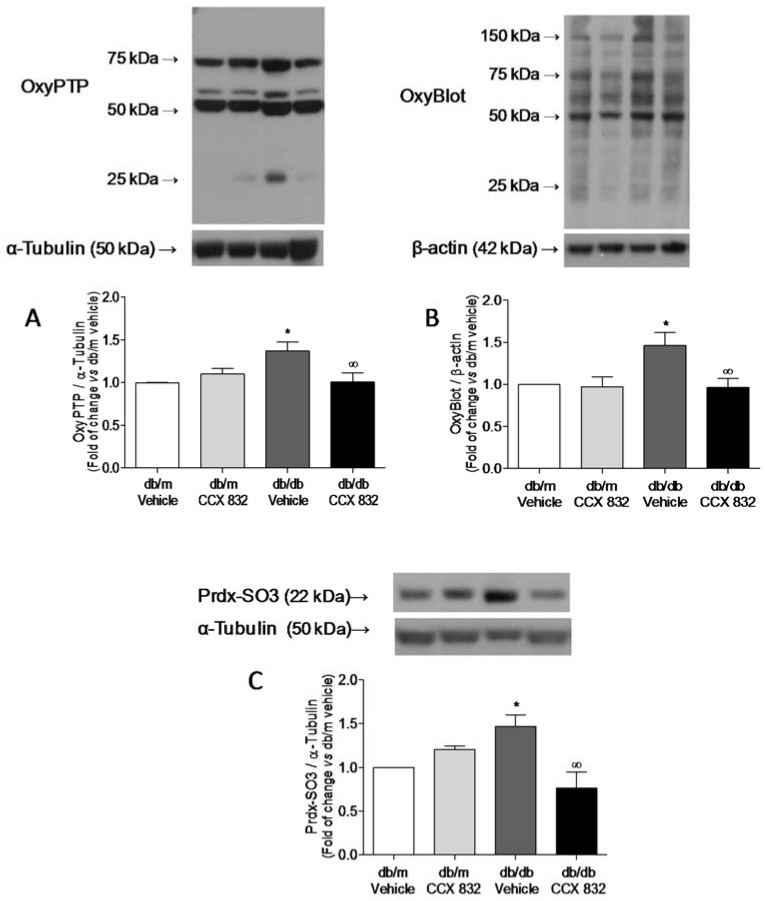
ChemR23 antagonism decreases renal carbonylation, protein-tyrosine phosphatases (PTPs) and Prdx oxidation in db/db mice. The experiments were performed in kidneys from vehicle or CCX832-treated db/m and db/db mice. (**A**) PTPs oxidation was assessed by western blot. (**B**) Protein carbonylation was assessed by OxyBlot. (**C**) Prdx-SO_3_ oxidation was assessed by western blot. The values were normalized by α-tubulin or β-actin expression. Bars represent the mean ± SEM of 5 to 8 experiments. * *p* < 0.05 vs. db/m, ∞ *p* < 0.05 vs. db/db vehicle.

Considering that (1) ROS Considering generation modulates many vascular processes via oxidation of proteins; (2) there is increased ROS production in kidneys from db/db mice; and (3) protein tyrosine phosphatases (PTPs) are particularly sensitive to oxidative stress and vulnerable to oxidation, which results in the inhibition of the enzymes, we addressed whether CCX832 interferes with renal PTP oxidation. In kidneys from db/db mice, PTP oxidation (Figure 3A) as well as peroxiredoxin oxidation (Prdx-SO_3_) and carbonylation, a type of irreversible oxidation induced by oxidative stress (Figure 3B,C, respectively) were significantly increased. Increased oxidation and carbonylation of proteins was not observed when the animals were treated with CCX832 (Figure 3A–C), showing that ChemR23 blockade plays a protective role in renal protein oxidation in db/db mice.

### 2.4. ChemR23 Antagonism Restores the Function of Renal Nrf2 Antioxidant System in db/m Mice

**Figure 4:**
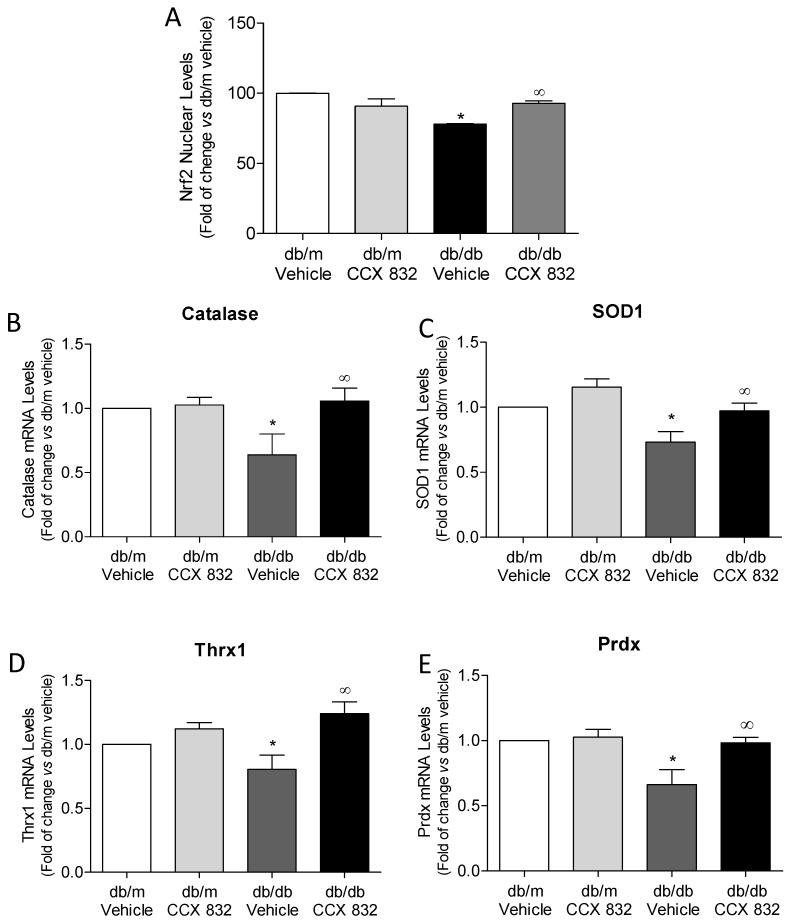
ChemR23 antagonism attenuates impaired Nrf2 nuclear translocation and increases antioxidant enzymes expression in kidneys from db/db mice. (**A**) Nuclear accumulation of Nrf2 was determined by ELISA in nuclear extract of kidneys from vehicle and CCX832-treated db/m and db/db mice. (**B**–**E**) mRNA expression of genes regulated by Nrf2 was determined by real time PCR. The values were normalized by total protein levels (**A**) or by GAPDH mRNA expression (**B**–**E**). Results represent the mean ± SEM of 5 to 8 experiments. * *p* < 0.05 vs. db/m, ∞ *p* < 0.05 vs. db/db vehicle.

To investigate the effects of CCX832 on Nrf2-regulated antioxidant enzymes in kidneys from db/db mice, we assessed Nrf2 activity and antioxidant enzymes mRNA expression. In addition to its effects on ROS generation and protein oxidation, *CCX832* also restored the impaired Nrf2 nuclear translocation (Figure 4A) observed in kidneys from db/db mice. Furthermore, CCX832-treated db/db mice did not exhibit decreased mRNA levels of catalase, superoxide dismutase-1 (SOD1), thioredoxin-1 (Thrx1) or peroxiredoxin-1 (Prdx1) as observed in kidneys from obese/diabetic mice (Figure 4B–E, respectively). CCX832 did not change Nrf2 translocation or antioxidant enzymes gene expression in db/m mice. These data suggest that ChemR23 antagonism improves renal Nrf2 antioxidant system in db/db mice.

**Figure 5:**
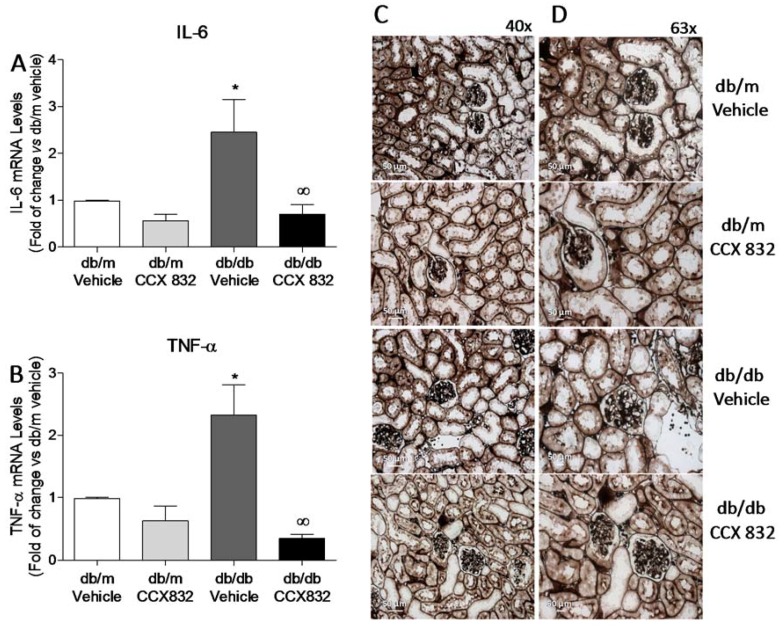
ChemR23 antagonism reduces the expression of renal inflammatory markers but does not change kidney structure in db/db mice. (**A**,**B**) mRNA expression, determined by real time-polymerase chain reaction (PCR), of IL-6 and TNF-α, respectively. The values were normalized by GAPDH mRNA expression. (**C**,**D**) Representative images of renal tubule and glomerulus stained with Grocott-Gomori’s methenamine-silver nitrate from vehicle and CCX832-treated db/m and db/db mice. Scale bar: 50 µm. Magnification of 40X (**C**) and 63X (**D**). Results represent the mean ± SEM of 4–5 experiments. * *p* < 0.05 vs. db/m, ∞ *p* < 0.05 vs. db/db vehicle.

In order to investigate the renal inflammatory profile of db/db mice, gene expression of two pro-inflammatory markers were addressed. Kidneys from db/db mice presented increased interleukin-6 (IL-6) and tumor necrosis factor-α (TNF-α) mRNA expression, which was attenuated by CCX832 (Figure 5A,B). Despite the high oxidative state and increased expression of inflammatory markers observed, no histological alterations were found at this stage in kidneys from db/db mice (Figure 5C,D), which indicates that the redox changes and inflammatory renal profile seen in db/db mice precede any renal structural abnormalities associated with the diabetic condition.

## 3. Discussion

Our knowledge on the chemerin/ChemR23 axis in cardiovascular function has rapidly progressed. Growing evidence indicates a role for chemerin in kidney disease. However, implications of chemerin in diabetic nephropathy are unclear. Here we advance the field by demonstrating an important role for chemerin in renal oxidative stress and renal dysfunction in a model of diabetes. Particularly, we show that chemerin receptor blockade by CCX832 reduces renal ROS production and protein oxidation. Potential mechanisms underlying the renal effects of chemerin involve upregulation of Noxs and downregulation of Nrf2-regulated anti-oxidant genes. These phenomena, observed in the absence of renal structural dysfunction or fibrosis, highlight that chemerin-induced renal alterations precede renal damage in this animal model.

Obesity and diabetes are major co-morbidities associated with cardiovascular complications, nephropathy and end stage-renal disease. Within the context of nephropathy, adipokines, such as leptin, resistin, visfatin and chemerin, have their serum levels markedly correlated to the disorders observed in renal diseases [41]. In the last few years, several reports have investigated the circulating chemerin levels and their potential significance to renal diseases development and progression. The interplay between chemerin and renal function was first demonstrated in chronic hemodialysis (HD) patients, in which serum chemerin levels were more than two-fold higher [42]. Later findings showed that serum creatinine is also associated with serum chemerin levels in type 2 diabetes patients [43]. Corroborating this, major findings in the present study demonstrate that ChemR23 blockade reduces albuminuria as well as urinary creatinine levels in diabetic obese mice.

Vascular and renal oxidative stress have been consistently demonstrated in experimental models [44,45,46] and patients with diabetes [47]. High glucose and other pro-diabetic conditions are known to increase ROS production via activation of Noxs [47,48]. In renal pathobiology Nox 4 is particularly important due to its abundance in the kidney [28] and it is a major source of ROS in renal cells and tissues during diabetes [49]. In line with these findings, we have demonstrated in this study that the db/db diabetic obese mouse model presents increased ROS generation and Nox 4 expression in kidneys, which were reversed by chemerin receptor antagonism. It is of importance to note that our group already demonstrated that chemerin impairs vascular function and signaling through Nox activation in human cells [33]. Although CCX832 has been previously described as a ChemR23 antagonist and a blocker of chemerin signals [4,50,51,52,53], we have not provided direct evidence that CCX832 disrupts chemerin/ChemR23 signaling, which represents a limitation of the present study.

Renal functional and morphological alterations during diabetes are ameliorated by antioxidant treatment [54,55]. Recent clinical studies evidenced a significant improvement in renal function in patients with advanced CKD and T2D treated with an oral antioxidant, bardoxolone [56]. Renal oxidative stress observed in db/db mice in this study was associated with decreased activity of Nrf2 as evidenced by its decreased nuclear translocation. Loss of Nrf2 activity was associated with downregulated expression of anti-oxidant genes, such as catalase, SOD1, thrx1 and prdx, which likely dampens the antioxidant defense in the kidneys from these animals. Once again, a key role of the chemerin/chemR23 system to renal oxidative stress in db/db mice was evidenced, since the blockade of ChemR23 reversed such deleterious responses.

ROS modulate several cellular responses via protein oxidation, such as inhibition of PTPs via oxidation of its cysteine residues [57]. In this study, the potential of irreversible oxidation was used to address a potential effect of chemerin in the regulation of PTP function. ChemR23 antagonism reduced the increased PTPs oxidation observed in kidneys from db/db mice, an effect concomitantly linked to decreased ROS generation in these tissues. The inactivation of PTPs in kidney may increase/augment phosphorylation of various signaling proteins, which can contribute to diabetes-associated renal dysfunction. Additionally, kidneys from db/db mice treated with CCX832 present a reduction in protein carbonylation, a biomarker for oxidative stress–induced irreversible damage that leads to loss of protein function [58]. Peroxiredoxin was also targeted by chemerin. Peroxiredoxin, when hyperoxidized, becomes inactivated [59], which makes the kidney vulnerable to damage associated with oxidative stress. These proteins may represent targets of the chemerin/ChemR23 system and may underlie their association with impaired renal function in diabetic obese mice.

Despite the increased renal mass, albuminuria, increased urinary creatinine levels and renal oxidative stress observed in db/db mice, no glomerular lesions, mesangial matrix expansion or alterations in fibrotic markers expression were observed in the kidneys of db/db mice. These data indicate that renal injury and functional changes observed in our study precede the structural damage in this animal model. A recent study showed that 10 weeks-old male db/db mice present a significant increase in urinary albumin and kidney weight although only a mild accumulation of mesangial matrix and no remarkable changes in the tubulointerstitium were found [60]. Cohen et al. [61] observed no changes in the mesangial compartment of the db/db mouse at 8 weeks of age although a twofold increase in mesangial matrix was found when the animals reached 12 weeks. Interestingly and corroborating the suggestion that adipokines play an important role in renal dysfunction during diabetes, a very recent study has shown that an orally administered synthetic adiponectin receptor agonist, which is an adipokine with protective effects during diabetes and metabolic syndrome through its anti-inflammatory, antifibrotic, and antioxidant effects, reverses diabetic nephropathy in 16 weeks-old male db/db mice [62].

## 4. Materials and Methods

### 4.1. Animals

All experimental protocols were performed in accordance with the Ethical Principles in Animal Experimentation adopted by the National Council for Animal Experimentation Control (CONCEA) and were approved by the Ethics Committee on Animal Use (CEUA; protocol 062∕2012—25 June 2012) of the University of Sao Paulo, Ribeirao Preto, Brazil and by the West of Scotland Research Ethics Service (70∕9021). Ten to twelve weeks-old male C57BL/6J, lean non-diabetic db/m and obese diabetic db/db mice were housed in individually cages in a room with controlled humidity and temperature (22–24 °C), and light/dark cycles of 12 h. Animals had free access to food and tap water. Mice were treated with vehicle (PEG400/cremophor) or CCX832 (a gift from ChemoCentryx, Inc., Mountain View, CA, USA), a ChemR23 antagonist (75 mg/kg/day, for 3 weeks, by oral gavage). Previous radiolabeled binding studies revealed that CCX832 binds to human, mouse and rat ChemR23 receptors with a low nanomolar affinity [4,51]. CCX832 was specially formulated by ChemoCentryx for the in vitro studies and for the daily oral administration in the in vivo studies. Body weight was measured weekly from the beginning of the treatment. Animals were separated into 4 groups: db/m + vehicle, db/m + CCX832, db/db + vehicle and db/db + CCX832. In initial experiments, two additional groups were included: db/m and db/db mice without any treatment for the same three week-period, or untreated db/m and db/db mice, respectively. Since no difference was observed between untreated and vehicle groups, the remaining protocols were performed in animals treated with vehicle and CCX832. At the end of the treatment, urine and kidneys were collected. The kidneys were weighed, and the kidney mass values were normalized by the respective tibia length. To respect the principles of the 3Rs (replacement, reduction and refinement) in research, we examined mice that we have previously characterized [63].

### 4.2. Assessment of Urinary Creatinine and Albumin Levels

Spot urine samples were collected immediately before death. Creatinine and albumin urine levels in vehicle- or CCX832-treated db/m and db/db mice were determined by an enzymatic reaction according to instructions from the manufacturer (Catalogue #127 and #19, respectively—Labtest^®^, Santa Barbara d’Oeste, Brazil).

### 4.3. Immunoblotting

Western blotting was used to evaluate protein expression in kidneys from db/m and db/db mice treated with vehicle or CCX832. Briefly, tissues were homogenized in lysis buffer [(in mmol/L) sodium pyrophosphate 50, sodium Fluoride (NaF) 50, sodium chloride (NaCl) 5, ethylenediaminetetraacetic acid (EDTA) 5, ethylene glycol tetraacetic acid (EGTA) 5, 4-(2-hydroxyethyl)-1-piperazineethanesulfonic acid (HEPES) 10, Sodium orthovanadate (Na_3_VO_4_) 2, phenylmethylsulfonyl fluoride (PMSF) 50, Triton 100 0.5%, and leupeptin/aprotinin/pepstatin (1 mg/mL)] and then sonicated for 5 s. Proteins extracted from each lysate were separated by electrophoresis on 8 to 12% sodium dodecyl sulfate (SDS) polyacrylamide gel and transferred to a nitrocellulose membrane for Western blotting with specified antibodies. Nonspecific binding sites were blocked with 5% milk in Tris-buffered saline solution with Tween for 1 h at room temperature. After incubation with secondary antibodies, signals were revealed with chemiluminescence, visualized by autoradiography, and quantified densitometrically with the open-source software ImageJ (available online: http://imagej.nih.gov/ij/). Results were normalized to β-actin or α-tubulin. Antibodies used were as follows: Prdx-SO_3_ (Abcam ab16830; 1:2000, Cambridge, MA, USA), OxyPTP (R&D Systems MAB2844; 1 µg/mL, Minneapolis, MN, USA), OxyBlot (Millipore S7150; 1:1000, Burlington, MA, USA), anti-α-tubulin and anti-β-actin are from Sigma (1:10,000, St. Louis, MO, USA).

### 4.4. Immunofluorescence

8-Hydroxyguanosine (8-OHG) is a modified guanosine that occurs in DNA/RNA due to attack by hydroxyl radicals that are formed as by products and intermediates of aerobic metabolism and during oxidative stress [64]. 8-hydroxyguanosine (8-OHG) immunohistochemistry has been widely used as a sensitive, stable and integral biomarker of oxidative stress-induced DNA and RNA damage.

Paraffin sections of kidney (3 µm) were deparaffinized in xylene, rehydrated through graded ethanol, and washed in water. All sections were incubated in EDTA (pH 8) and boiled for 15 min (min) at 95 °C for antigen unmasking. Slides were cooled to room temperature, permeabilized in 0.5% Triton X-100 in phosphate-buffered saline (PBS) for 5 min, and blocked with 10% donkey serum, 1% bovine serum albumin (BSA) in 1X Tris-buffered saline and Tween 20 (TBS-T) for 1 h at room temperature in a humidified chamber. For 8-OHG immunostaining, slides were incubated overnight with anti-8-OHG goat polyclonal antibody (Abcam ab10802, 1:200 diluted in 5% donkey serum, 0.02% BSA, 0.0025% Tween-20 in 1X TBS solution) in a humidified chamber. Alexa-fluor-488-conjugated donkey anti-goat secondary antibody (Molecular probes, A-11055, 1:300 dilution in 5% donkey, 0.02% BSA, 0.0025% Tween-20 in 1X TBS solution, St. Louis, MO, USA) was used after primary antibody incubation for 1 h at room temperature in the dark. The slides were treated with 0.1% Sudan Black B (Sigma Aldrich, Cat. Number: 199664, St. Louis, MO, USA) in methanol for 10 min to remove lipofuscin-mediated autofluorescence. Nuclei were counterstained with 4-6-diamidino-2-phenylindole (DAPI at 100 µg/mL) for 10 min. Sections were mounted with a coverslip using ProLong Gold anti-fade mounting media containing DAPI (Molecular probes, P-36931) in the dark. Fluorescence images were captured using Axiovert 200M microscope with a laser-scanning module LSM 510 (Carl Zeiss AG, Heidelberg, Germany). For negative controls, goat IgG-matched isotype controls were used (Santa Cruz, sc-2028, Dallas, TX, USA). The fluorescence intensity, which indicates DNA damage induced by oxidative stress, in the endothelium and SMC layers was measured.

### 4.5. Amplex Red Assay

Hydrogen peroxide (H_2_O_2_) levels were assessed in kidney according to the manufacturer’s instruction using the horseradish peroxidase-linked Amplex Red fluorescence assay (Life Technologies, Carlsbad, CA, USA).

### 4.6. Real Time PCR

Quantitative real-time PCR (Applied Biosystems, Foster City, CA, USA) was used to analyze mRNA expression. Briefly, total RNA was extracted from kidneys using TRIzol (Qiagen, Manchester, UK), treated with RNase-free DNAse I, and 2 μg of RNA were reverse transcribed in a reaction containing 100 μg/mL oligo-dT, 10 mmol/L of 2′-deoxynucleoside 5′-triphosphate, 5X First-Strand buffer, and 2 μL of 200-U reverse transcriptase. For real-time PCR amplification, 3 μL of each reverse transcription product were diluted in a reaction buffer containing 5 μL of SYBR Green PCR master mix and 300 nmol/L of primers in a final volume of 10 μL per sample. The reaction conditions consisted of 2 steps at 50 °C for 2 min and 95 °C for 2 min, followed by 40 cycles of 3 steps, 15 s denaturation at 95 °C, 60 s annealing at 60 °C, and 15 s at 72 °C. The relative mRNA expressions (target gene/Gapdh housekeeping gene) were calculated by ΔΔ*C*t method. The following mice primers were used in this study: Nox 1 (FW: TCCCTTTGCTTCCTTCTTGA; RW: CCAGCCAGTGAGGAAGAGTC), Nox 2 (FW: CGCCCTTTGCCTCCATTCTC; RW: CCTTTCCTGCATCTGGGTCTCC), Nox 4 (FW: CCAGAATGAGGATCCCAGAA; RW: AGCAGCAGCAGCATG); Catalase (FW: ACATGGTCTGGGACTTCTGG; RW: CAAGTTTTTGATGCCCTGGT), SOD1 (FW: GAGACCTGGGCAATGTGACT; RW: TTGTTTCTCATGGACCACCA), THRX1 (FW: TGTCGTGGTGGACTTCTCTG; RW: TCAAGGAACACCACATTGGA) and PRDX1 (FW: CACCCAAGAAACAAGGAGGA; RW: AAAAAGGCCCCTGAAAGAGA).

### 4.7. Protein Oxidation

Levels of protein-tyrosine phosphatases (PTPs) and peroxiredoxin oxidation were evaluated by Western blot and carbonylation by OxyBlot assay (Millipore S7150).

The OxyBlot assay performed in this study detects carbonyl groups introduced into proteins’ side chains by a site-specific mechanism. All bands in the membranes were quantified considering that many proteins undergo carbonylation and that the antibody is not specific for a particular PTP.

### 4.8. Nrf2 Activity

To determine nuclear accumulation of Nrf2, kidney nuclear lysates were separated using the Active Motif nuclear extract kit (Active Motif, Carlsbad, CA, USA) following the manufacturer’s protocol. Briefly, lysates were resuspended in 1X hypotonic buffer and centrifuged for 30 s at 14,000× *g* in a microcentrifuge pre-cooled at 4 °C. Nuclear pellets were resuspended in lysis buffer provided by the manufacturer. The suspension was incubated for 30 min on ice on a rocking platform set at 150 rpm and then centrifuged for 10 min at 14,000× *g*. The supernatant was transferred to a prechilled microcentrifuge tube. TransAM Nrf2 ELISA kit (Active Motif) was used to measure nuclear accumulation of Nrf2 at a wavelength of 450 nm.

### 4.9. Histopathology

Kidneys were removed and weighed. The kidney weight (g) was corrected by the length of the tibia (mm). Kidneys from control and treated animals were fixed in 4% neutral buffered formalin solution, rinsed with 70% ethanol, dehydrated through a graded alcohol series, and embedded in paraffin. Sections of 3 μm of renal tissue from these animals were stained methenamine-silver nitrate for histological analysis. For each stained section, the glomerular mesangial expansion and tubulointerstitial injury were evaluated using scores on a scale of 0 to 4 that reflected changes in the extent (0 = 5–25%, 1 = 25–50%, 3 = 50–75%, and 4 = 75–100%) of renal injury/ renal abnormalities. The Zeiss Axio microscope with AxioCam MRC (Program AxioVision Release 4.8.2. [06-2010], Oberkochen, Germany) was used for images acquisition.

### 4.10. Statistical Analysis

Statistical analysis was performed using GraphPad Prism 3.0 (GraphPad Software Inc., San Diego, CA, USA). Data are presented as means ± standard error of the mean (SEM). Groups were compared using student’s *t* test or one-way analysis of variance (ANOVA). Bonferroni or Tukey’s post-test were used as appropriate. Results of statistical tests with *p* < 0.05 were considered as significant.

## 5. Conclusions

In conclusion, our data suggest that chemerin plays a role in renal damage in diabetic mice through ROS- and oxidation-dependent mechanisms. CCX832 plays a renoprotective role by decreasing oxidative status during the early stages of diabetes, when structural alterations are not observed. These findings advance our understanding on potential pathways modulated by chemerin/ChemR23 axis, and their role in diabetic nephropathy. In this context, CCX832 may be a novel promising candidate to ameliorate diabetes-associated nephropathy.

## Supplementary Materials

Supplementary materials can be found at http://www.mdpi.com/1422-0067/19/8/2454/s1.

## Abbreviations

ChemR23	chemerin receptor
CCX832	chemerin antagonist
Nrf2	Nuclear Factor Erythroid Like 2
PTPs	protein tyrosine phosphatases
Prdx-SO_3_	peroxiredoxin oxidation
SOD1	superoxide dismutase-1
Thrx1	thioredoxin-1
Prdx1	peroxiredoxin-1
T2D	type 2 diabetes
CKD	chronic kidney disease
ROS	reactive oxygen species
Nox	Nicotinamide adenine dinucleotide phosphate (NADPH) oxidase
8-OHG	8-Hydroxyguanosine
H_2_O_2_	hydrogen peroxide
O_2_^−^	superoxide anion
