# Supplementary material for: Upregulation of Nrf2 and Decreased Redox Signaling Contribute to Renoprotective Effects of Chemerin Receptor Blockade in Diabetic Mice
Source: Int J Mol Sci. 2018 Aug 19;19(8):2454. doi: 10.3390/ijms19082454 (PMC6121242; doi:10.3390/ijms19082454)
Supplement: Supplementary file 1 [file ijms-19-02454-s001.pdf]

Supplementary Materials: Upregulation of Nrf2 and Decreased Redox Signaling Contribute to Renoprotective Effects of Chemerin Receptor Blockade in Diabetic Mice

Karla Bianca Neves, Augusto Cesar Montezano, Rheure Alves-Lopes, Thiago Bruder-Nascimento, Rafael Menezes da Costa, Roberto S Costa, Rhian M Touyz and Rita C Tostes

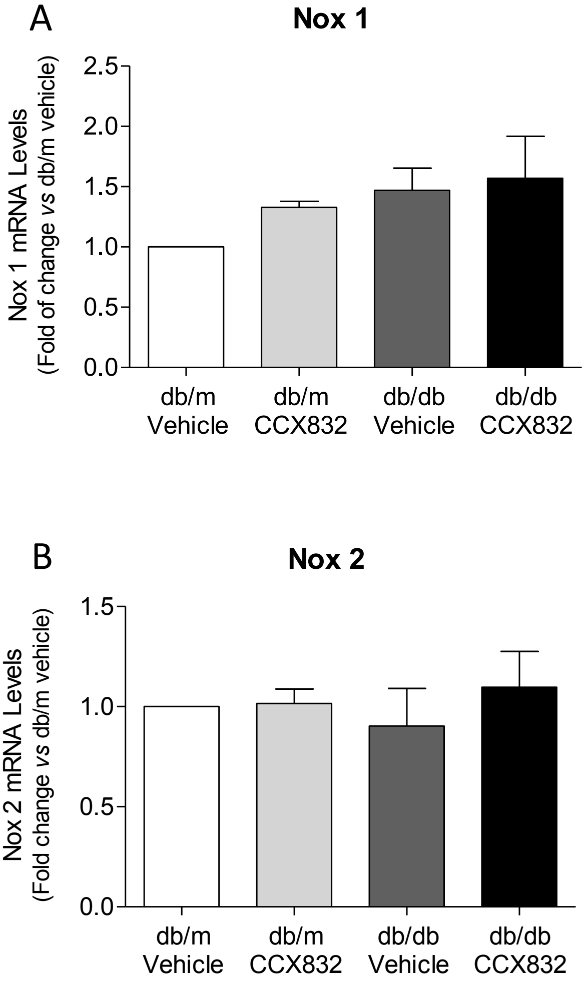

Figure S1. ChemR23 antagonism does not change Nox1 or Nox2 expression in kidneys from db/*db* mice. mRNA expression of Nox1 (**A**) and Nox2 (**B**) was determined by real time PCR. The values were normalized by GAPDH mRNA expression. Results represent the mean ± SEM of 5 to 6 experiments. .
